# The Paradox of Care: Burnout Among Healthcare Workers

**DOI:** 10.3389/phrs.2025.1609096

**Published:** 2025-10-23

**Authors:** Shevaun M. Teo, Sonja Moore, Shweta Rao

**Affiliations:** ^1^ Department of Nutrition and Dietetics, HSE Midland Regional Hospital Portlaoise, Portlaoise, Ireland; ^2^ Department of Public Health HSE Dublin and Midlands, Dublin, Ireland; ^3^ Immunization Bureau, Houston Health Department, Houston, TX, United States

**Keywords:** healthcare worker burnout, workforce wellbeing, health system resilience, multidisciplinary team, occupational stress

## Introduction

Burnout is often defined as unsuccessfully managed chronic stress from work [1] and is a widespread issue amongst healthcare workers (HCWs). Recent meta-analyses report prevalences ranging from over 70% among nurses and doctors to 56% in dietitians, 43% in emergency staff, and 40% in midwives [2]. Within the HSE (Health Service Executive; Ireland’s largest employer and main deliverer of health services), the pattern is equally concerning: a 2024 occupational health survey of acute and community services found burnout symptoms in 70% of respondents [3]. While this and similar cross-sectional studies provide essential prevalence data, they often overlook the deeper, structural roots of burnout.

A growing body of research highlights how chronic understaffing, compounded by poor institutional support, drives sustained stress and burnout among healthcare workers [4]. Staff shortages not only increase workloads and shift lengths but also erode job satisfaction and heighten moral distress when workers feel unable to provide safe care. In Ireland, these pressures were stark during the COVID-19 pandemic, when healthcare professionals across roles (not only physicians and nurses but also allied health professionals [AHPs], administrative staff, and non-frontline workers) were forced to rapidly adapt to new systems under significant pressure.

## Burnout Drivers and HCW Experiences

Burnout is common among junior doctors, with many reporting having time to do little besides work and sleep, leaving no space for hobbies, healthy routines, or personally fulfilling activities [5]. A significant contributing factor is the lack of flexibility in rostering, with no slack for sudden changes such as unplanned sick leave. Anecdotally, junior doctors have been pressured by HR to arrange their own cover when calling in sick. This discourages necessary leave and leads to presenteeism, which poses direct risks to patient safety. Covering for absent colleagues typically falls on the remaining staff, compounding fatigue and stress. Internationally, rostering stand-by staff and designated “floaters” has helped to cover sudden absences, but in Ireland such measures remain underused, with hospitals instead relying heavily on costly agency staff to fill roster gaps.

While much attention focuses on physicians and nurses, AHPs such as physiotherapists and dietitians also face significant risks of burnout and workforce shortages. A recent rapid review by Roth et al. [6] highlighted that AHP shortages are widespread internationally, leading to longer waiting times, reduced continuity of care, and poorer health outcomes. These findings underscore that healthcare systems cannot function effectively without adequately staffed AHP roles, which play an essential role in chronic disease management, rehabilitation, and patient recovery. Ensuring their retention and wellbeing is therefore central to workforce planning and to reducing burnout across the health system as a whole.

Alongside staffing shortages, dissatisfaction with administrative systems further erodes morale. Junior doctors frequently report disrespectful treatment by hospital HR and payroll departments, including delayed salary payments and non-payment for approved overtime. Long and antisocial working hours persist despite reductions in 24-hour call, and new policy proposals to expand weekend coverage have not been matched with sufficient recruitment. The result is a system that expects staff to “do more with less,” further exacerbating burnout.

Finally, a factor that remains underdiscussed is the trauma inherent to healthcare work. Many HCWs face exposure to suffering, death, and effects of violence, sometimes directed at them - particularly nurses and paramedics. Junior doctors report being left to make complex clinical decisions beyond their training without adequate senior support [5]. The psychological burden associated with responsibility in life-or-death situations is an under-recognised determinant of occupational stress and burnout. Despite acknowledgement of the value of psychological debriefing, the absence of formalised structures means it occurs inconsistently and without reliability. Although an Employee Assistance Service is available, staff awareness of it is limited and it is often perceived as a last resort, accessed only when experiencing severe distress.

Alongside rising patient ratios, inefficiencies in referral and administrative processes add to stress across the multidisciplinary team. For example, AHPs often receive inappropriate or incomplete referrals which, even when declined, require time-consuming processing and communication. Ireland’s lagging digital landscape in healthcare, as evidenced by being ranked last in many indicators of digital health readiness [7], adds to inefficiencies and staff frustration in hospitals. Together, such inefficiencies highlight the need for system-level interventions that support all professions, not only physicians and nurses.

## Policy Responses Falling Short

A healthy work-life balance is key for burnout prevention and management. [8]. The HSE purports to offer a variety of work-life balance initiatives such as the right to disconnect, blended and flexible working and a shorter working year. However, in practice, many of these schemes do not apply to clinical work, or HCWs in competitive clinical training do not feel they can avail of such schemes in fear of risking career progression.

Despite the prevalence of burnout among HCW, the “Staff Health and Wellbeing” section of the HSE website makes no mention of burnout, and the resources surrounding mental wellbeing focus on positive psychology and mindfulness-based approaches, emphasising an individual’s resilience, defined as “the ability of an individual to cope with and adapt positively to adversity” as a determinant of their wellbeing.

The emphasis of resilience training as a solution to staff burnout has long been panned by HCWs, arguing that relying on staff resilience alone in the absence of any structural and systemic change is an incomplete intervention [9].

## Recommended Solutions

Addressing healthcare worker burnout necessitates real commitment from leaders to analyse and remediate its root causes systematically. Work-life balance must be recognised as a critical determinant of staff wellbeing and actively supported through measures such as adherence to work-time limits, adequate rostering redundancy to absorb absences, and the facilitation of leave. Not all solutions require additional hiring; alternative models of working, such as ward-based rostering or specialty-based patient cohorting, can improve efficiency and reduce stress, although implementation in Irish hospitals has often been constrained by chronic bed shortages. Equally, AHPs require sustainable caseloads and streamlined referral pathways to minimise avoidable administrative burden. Time should be specifically allocated for discussion of staff wellbeing and formal opportunities for psychological debriefing.

## Conclusion

While the authors’ experiences come mainly from Ireland, these issues transcend borders, as many health systems face the same “do more with less” pressures at the expense of HCW wellbeing. Leaders must recognise that investment in staff wellbeing is not a cost but a prerequisite for sustainable productivity. Burnout is not simply frontline fatigue but a symptom of broader systemic failure, contributing to diminished care quality, absenteeism, and turnover. Yet most research remains limited to prevalence studies that show the scale of the problem but not its causes. More qualitative work is urgently needed to capture lived experiences and guide meaningful, system-level solutions. Without this, policy responses will continue to treat symptoms rather than root causes, thus placing the burden of resilience back on already overstretched staff.

What emerges is a troubling paradox: the very systems designed to preserve health are undermining the health of their own workforce. Unless this contradiction is acknowledged and addressed through structural reform, policy responses will continue to treat symptoms rather than causes - and the vicious cycle of burnout will persist, to the detriment of both staff and patients.
